# The quality of patients’ self-blood pressure measurements: a cross-sectional study

**DOI:** 10.1186/s12872-021-02351-5

**Published:** 2021-11-12

**Authors:** Katarzyna Nessler, Anna Krztoń-Królewiecka, Anna Suska, Mitchell R. Mann, Michał B. Nessler, Adam Windak

**Affiliations:** 1 Department of Family Medicine, Jagiellonian University Medical College, Bocheńska 4, 31-061 Kraków, Poland; 2 Department of Family Medicine, Students’ Family Medicine Interest Group, Jagiellonian University Medical College, Kraków, Poland; 3 Burns and Plastic Surgery Centre of Malopolska, Burns and Plastic Surgery Centre of Malopolska, Rydygier Memorial Hospital, Rydygier Memorial Hospital, Os. Zlotej Jesieni 1, 31-826 Kraków, Poland

**Keywords:** Home blood pressure monitoring, HBPM, Hypertension management

## Abstract

**Background:**

The accurate and independent measurement of blood pressure (BP) by patients is essential for home BP monitoring (HBPM) and determining the quality of hypertension (HTN) control. This study aimed to evaluate the BP self-measurement techniques of hypertensive patients and their accuracy in accordance with established guidelines. We sought to identify the common errors that patients make and suggest improvements that can be implemented in the primary healthcare setting to increase the reliability of HBPM conducted by hypertensive patients.

**Methods:**

One hundred patients diagnosed with HTN completed a questionnaire inquiring about their health and demographic data and BP monitoring practices. Patients were then observed and filmed while measuring their BP on their own devices in five primary healthcare centres in Kraków, Poland. The correctness of their techniques was assessed in accordance with the European Society of Hypertension guidelines on HBPM.

**Results:**

Only 3% of patients measured their BP without error; 60% made three or more errors. The most frequent error, made by 76% of subjects, was incorrect sphygmomanometer cuff placement (above or below heart level, or/and the indicator mark was not aligned with the brachial artery). Regarding patients’ previous instruction for the correct use of their devices, 36% of patients referred to their monitor’s user manual, 22% did not receive any prior assistance, and only 29% were adequately counselled by physicians on how to measure their BP correctly.

**Conclusions:**

Our findings suggest that primary healthcare physicians and their personnel often do not adequately instruct patients on how to measure their BP correctly. Therefore, healthcare systems must provide patients with more adequate training and reference materials on the best practices of BP monitoring.

**Supplementary Information:**

The online version contains supplementary material available at 10.1186/s12872-021-02351-5.

## Background

Hypertension (HTN) is one of the principal risk factors for cardiovascular disease and is responsible for the deaths of approximately nine million people annually worldwide [1]. The global prevalence of HTN amongst adults is 30–45%; in Poland, it is approximately 32% [2, 3].

Reliable blood pressure (BP) measurements are critical to the effective diagnosis and treatment of HTN. Compared with those of office BP measurement (OBPM), BP values obtained via home BP monitoring (HBPM) are typically lower [4].

HBPM is the average of all BP readings performed with a semiautomatic, validated BP monitor for at least three days and preferably six to seven consecutive days. Readings should be taken in the mornings and evenings in a quiet room after five minutes of rest and in a seated position with the back and arm supported [5]. Two measurements, one to two minutes apart, should be taken each time [5].

HBPM has been shown to provide more reproducible data than OBPM [4]. Recent meta-analyses show that HBPM-based treatments are strongly recommended in the control of HTN [6, 7]. Furthermore, recent studies have indicated that BP measurements made by patients at home better predict cardiovascular morbidity and mortality [8]. BP self-monitoring has also been shown to have a beneficial effect on medication adherence and BP control [9, 10]. However, it has been observed that even minor systematic errors in BP measurements may cause substantial variations in the proportion of patients being diagnosed with HTN [11, 12]. Moreover, this relatively simple technique is prone to numerous patient-caused errors that may significantly distort measurement results.

This study aimed to answer the following questions:

1. Do patients measure their BP in accordance with the standards set up by the European Society of Hypertension (ESH) guidelines [13]?
2. What are the most common errors made by patients when measuring their BP?
3. What sources of information about correct BP measurement techniques do patients use?
4. Are there any associations between errors made by patients during self-measurement of their BP and their personal characteristics?

## Methods

### Study design

This cross-sectional study was conducted between July 2016 and May 2018. Participants were recruited from five primary healthcare centres in the Kraków area in southern Poland, which agreed to participate in the study. Medical students from Jagiellonian University Medical College in Kraków were trained to act as research fieldworkers. They checked the inclusion criteria of patients who agreed to participate, obtained their informed consent, asked them to fill in the study questionnaire, and filmed their BP self-measurement routines. Study participants brought their own BP gauges and used them while demonstrating their BP self-measurement techniques. This study was approved by the Jagiellonian University Bioethics Committee (122.6120.121.2015; 25 June 2015) and was conducted according to good clinical practice (GCP) rules.

### Sampling and study participants

The minimum patient sample size (*n*) calculated with OpenEpi software was estimated to be 97 (detailed information in Additional file 1, Additional file 2). One hundred forty-seven patients were approached as potential subjects in the order in which they applied for a medical appointment for any reason. The study participants were 100 consecutive patients who fulfilled the inclusion criteria and agreed to take part in the study (response rate: 68%). The inclusion criteria were: (1) age of 18 or older; (2) diagnosed HTN in accordance with ESH guidelines, defined as an office systolic BP ≥ 140 and/or diastolic BP ≥ 90 mmHg [13]; (3) declared regular BP monitoring at home; (4) a lack of current or past arrhythmias; and (5) a lack of comorbidities that could prevent effective communication (e.g., cognitive, visual, or hearing impairments, motor difficulties, inabilities to give informed consent). No restrictions were implemented to select for patients’ level of BP monitoring training.

### Measurements

The questionnaire had two parts and consisted of 27 questions (Additional file 1, Additional file 2). Part one collected basic patient demographic and medical history data, while part two collected information on patient knowledge of HBPM techniques.

Patients sat comfortably in a quiet environment for five minutes and were then asked to measure their BP using their sphygmomanometers in the same way that they would at home. Each patient completed two BP measurements one to two minutes apart. A third measurement was performed if the first two readings differed by > 10 mmHg. BP values were recorded as the average of the last two readings. Patients were filmed for all proceedings and were aware of their surveillance throughout. Five minutes after the final BP measurement made by each patient, a researcher conducted a BP measurement with an upper arm automatic sphygmomanometer.

Two independent observers reviewed the footage of each patient and assessed its accordance with the 2010 ESH guidelines for HBPM [13]. In the case of disagreements between the two observers, a third independent opinion was sought for arbitration.

### Statistical analysis

Statistical analyses were performed with Statistica 13.3 software (TIBCO Inc.). To present the results, we used descriptive statistics. To investigate the associations between specific errors made by patients and their characteristics, the Chi-square and Mann-Whitney U tests were performed for qualitative and quantitative variables, respectively. To analyse the associations between the number of errors made and patient characteristics, we used the Mann–Whitney U test, the Kruskal–Wallis test, and Spearman’s rank correlation coefficient. Multiple linear regression analysis was used to measure the final associations between patient characteristics and the number of errors made, adjusted for covariates. An alpha value of *p* = 0.05 was considered statistically significant.

## Results

### Respondent characteristics

One hundred patients with a mean age of 66.19 years (SD = 10.07 years; range: 36–85 years) with diagnosed HTN were recruited. The sample consisted mostly of female patients (61%) and those from a city of over 50,000 inhabitants (69%). Forty-one per cent had a 1st to 3rd level European Qualifications Framework (EQF) education, 34% had a 4th to 6th level education, and 25% had a 7th or 8th level completed [14]. The average time from HTN diagnosis to the present study was 12.5 years (SD = 8.24 years; range: 1–32 years). The mean patient body mass index (BMI) was 29.95 kg/m^2^ (SD = 4.76 kg/m^2^; range: 19.37–42.25 kg/m^2^). Sixty-three per cent of patients had a family history of HTN; 29% of patients had coexisting chronic diseases.

Most patients used an upper arm automatic sphygmomanometer (64%), 11% used aneroid devices, and upper arm semi-automatic and wrist gauges were used by 7% and 18% of patients, respectively.

### Sources of information about correct BP self-measurement

The main source of information on BP self-measurement techniques was the sphygmomanometer user manual (36%). Of the 19 patients given information by a general practitioner, 18 received oral instructions (11 observed a live demonstration). Ten patients were instructed by their cardiologist. One in five patients did not receive any information on how to measure their BP properly.

### Accuracy of BP self-measurement

Only 3% of patients made no errors while recording their BP. Sixty per cent of patients made three or more errors; the most frequent one, made by more than three-quarters of the study participants, was incorrect cuff placement (above or below heart level and/or the indicator mark was not aligned with the brachial artery). Seventy per cent of patients did not support their back during BP self-measurements. Additionally, the upper limb of 56% of patients was incorrectly placed. Other patient errors included: not being in a seated position (1%), holding a conversation during measurements (8%), failing to lay their fingers loosely (14%), keeping their legs crossed (20%), wearing clothing that compressed the shoulder region (22%), and the incorrect placement of the cuff (27%).

**Fig. 1:**
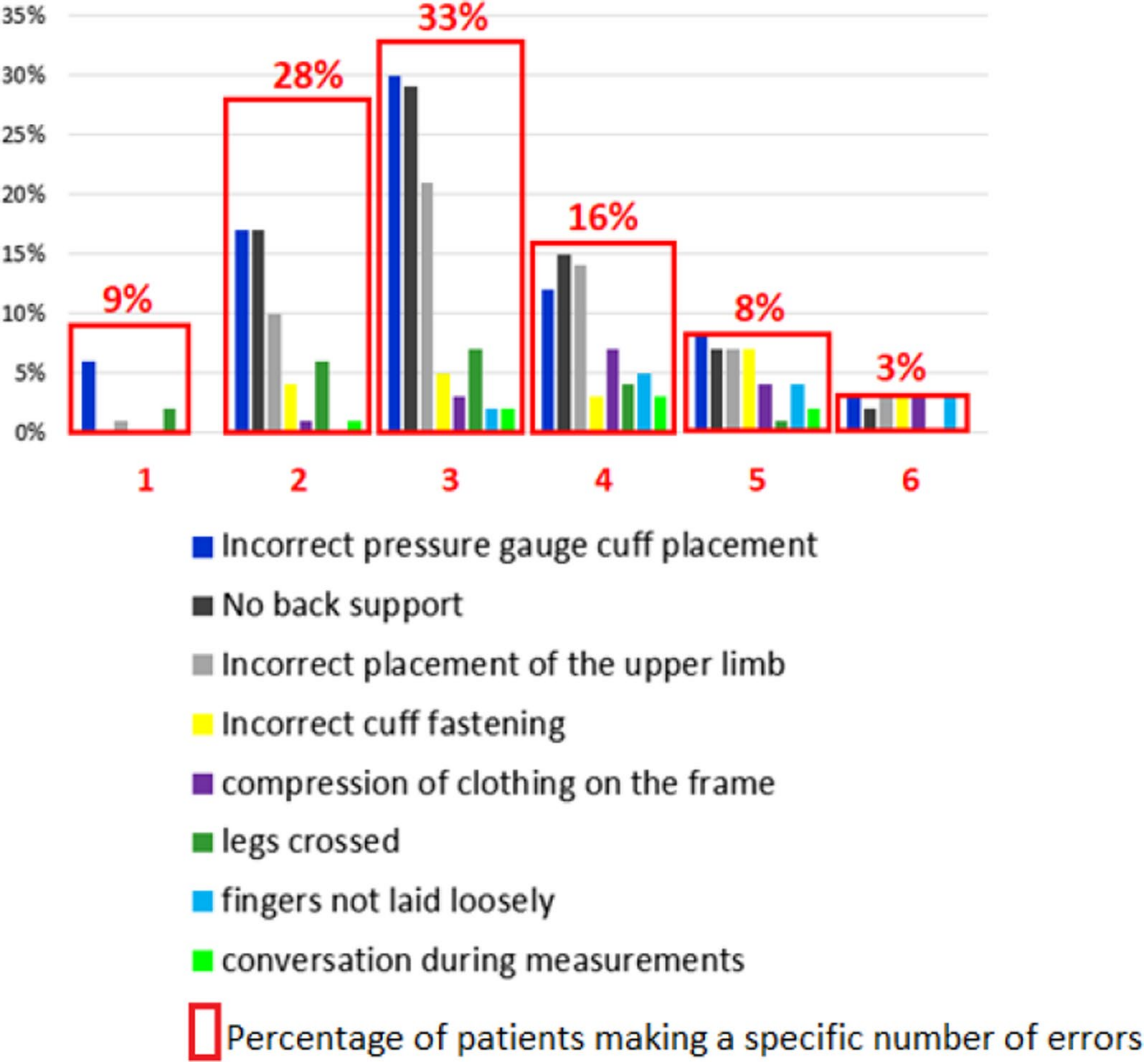
Number and types of errors made by patients during BP self-measurement

Figure 1 Number and types of errors made by patients during BP self-measurements.

### Type of errors and patient characteristics

Older patients were most likely to lack back support (*p* = 0.042), while those with a higher BMI were more likely to incorrectly fasten the cuff (*p* = 0.014) and not relax their fingers during testing (*p* = 0.013). Associations between patient characteristics, sphygmomanometer type, and particular errors are presented in Table 1.

**Table 1:** Associations between patients’ characteristics, sphygmomanometer type and measurement errors

Characteristics of the patients	Type of errors				
	Lack of back support	Incorrect upper limb position	Incorrect cuff fastening	Fingers relaxed	Talking during measurement
Gender			*p* = 0.023		
Female			36%		
Male			12.9%		
Education					*p* = 0.037
1st–3rd EQF level					4.88%
4th–6th EQF level					2.94%
7th–8th EQF level					20.00%
Place of living	*p* < 0.001				
Village or town of less than 50,000 inhabitants	38.71%				
City of more than 50,000 inhabitants	84.06%				
Family history of hypertension				*p* = 0.016	*p* = 0.020
Yes				20.63%	3.17%
No				2.70%	16.22%
Type of the sphygmomanometer		*p* < 0.001		*p* = 0.001	*p* = 0.008
Aneroid gauges		100%		27.27%	27.27%
Upper arm automatic		46.88%		10.94%	3.13%
Upper arm semi-automatic		14.29%		57.14%	28.57%
Wrist		77.78%		0%	5.56%

There was no observed dependence on errors regarding incorrect cuff position, pressure on the arm, or crossing the legs in relation to recorded patient characteristics.

### Number of errors and patient characteristics

Patients inhabiting villages and towns with fewer than 50,000 inhabitants showed significantly more errors in recording their BP than patients living in larger cities (p=0.002). Patients using automatic and wrist sphygmomanometers showed fewer errors than those using aneroid sphygmomanometers (p=0.005).

### Association between the number of BP self-measurement errors and patient characteristics—multiple regression analysis

Patients with longer HTN diagnoses made more errors than those diagnosed more recently (r_s _= 0.201; *p* = 0.045).

Multiple regression analysis results are shown in Table 2 (*p* for model=0.034; R^2^=0.216). Fewer errors were made by patients using upper arm (*p* = 0.020) and wrist (*p* = 0.007) automatic sphygmomanometers compared to aneroid sphygmomanometers.

**Table 2:** Multiple linear regression model: number of errors made during BP self-measurements associated with patient characteristics (reference group indicated in italics)

Patient characteristics		Beta	b	*p*
Variable	Comparison			
Gender				
* Female *	Male	− 0.147	− 0.387	0.179
Education				
* 1st–3rd EQF level *	4th–6th EQF level	− 0.106	− 0.287	0.331
	7th–8th EQF level	− 0.076	− 0.224	0.493
Place of living				
* Village or town of less than 50,000 inhabitants *	City of more than 50,000 inhabitants	0.188	0.521	0.093
Family history of hypertension				
*No*	Yes	− 0.056	− 0.148	0.606
Chronic comorbidities				
*No*	Yes	− 0.010	− 0.028	0.924
Type of sphygmomanometer				
*Aneroid*	Upper arm automatic	− 0.366	− 0.977	0.020
	Upper arm semi-automatic	− 0.106	− 0.531	0.421
	Wrist	− 0.414	− 1.381	0.007
Age		− 0.109	− 0.014	0.348
Time of hypertension diagnosis		0.157	0.024	0.153
BMI		− 0.045	− 0.012	0.700

## Discussion

### Summary of main findings

Only 3% of the participants measured their BP without errors; 60% made three or more errors. The most common errors were using an incorrect pressure-gauge cuff, a lack of proper back support, and incorrect cuff placement on the upper limb. Patients living in cities and those using upper arm automatic, or wrist-arm devices made fewer errors than those living in villages or small towns and using aneroid gauges. Only one-third of patients received instructions on proper BP measurement from a healthcare professional; 22% did not receive any instructions.

### Strengths and limitations

The methods used in this study regarding the recording and evaluation of BP self-measurement processes against standard measures are innovative.

However, this study presents some limitations. Firstly, the investigation was limited to one region of Poland, and the patients’ sampling was not random. Accordingly, the study results cannot be generalized to the entire population of patients checking their BP at home. The enrolled participants measured their BP daily and, compared to the average patient, had a greater understanding of their overall health and were often more compliant with recommendations for therapy, ultimately increasing treatment success rates [15]. Secondly, patients measuring their BP in the clinic might perform a different routine than at home as they may feel stressed by researchers observing and recording the proceedings. Thus, one can expect that they measured their BP with a greater than normal level of attentiveness.

Also, it is necessary to add that all participants were requested to wait 5 min before they measured their BP. This procedure was a part of the protocol; however, it is uncertain whether patients would have paid sufficiently attention to this aspect of self-measurement at homes.

It should be further noted that this study was conducted in accordance with the 2010 ESH guidelines for HBPM [13] before the implementation of the 2021 ESH guidelines [16]. However, the assessment of patient techniques for errors and the measurement techniques of the researchers were conducted in a manner also consistent with the new guidelines.

### Comparison with existing literature

A study by Wagner et al. showed that a third of their patients were unable to accurately self-record their BP and that none of the collected measurements met technique recommendations in their entirety. As in this study, patients showed inaccurate recording techniques and a lack of understanding of how to check their BP [17].

A’Court et al. assessed sphygmomanometer accuracy, finding digital devices to be as accurate as those with mercury and determining that aneroid monitors had higher failure rates [18]. In another study, the use of wrist devices led to frequent detection of falsely elevated BP values [19].

Comparisons of BP measurements performed via “pragmatic” (measurements taken hastily with lower regard for the correct protocol to save time, such as in hectic medical settings) and standardised (as per protocol) methods were analysed in a study by Mlawanda et al. [19]. It was concluded that these two forms of measurements were different and should not be clinically interchanged. The study revealed that 16.7% of patients had their treatment options misclassified and that the mean BP was 143/90 mmHg when measured pragmatically versus 133/87 mmHg when measured in a standardised fashion.

González-López et al. assessed the knowledge of BP measurement procedures amongst Spanish medical and nursing students and revealed that only 51.8% of them knew how to measure their BP correctly [20]. More knowledge was demonstrated amongst nursing students over medical ones. However, the findings of another study conducted in Australia indicated that nurses had an inadequate knowledge base for performing BP measurements in a standardised manner and failed to prevent introduced error [21]. The above observations further highlight that even medical personnel experience difficulties with proper BP measurement techniques, putting into question their ability to educate patients on the subject.

Contrary to our findings, Nordmann et al. reported that patients could be trusted in reporting BP measurements accurately but that those with a low educational background should receive ambulatory measurements [22].

A study by Li et al. revealed that inappropriate cuff placement as an isolated error did not significantly affect the accuracy of BP measurements [23].

### Implications for research and clinical practice

We interpret our results with caution. Nevertheless, the data suggest that most patients with HTN do not measure their BP correctly. This is not surprising as most subjects were never adequately counselled.

Although this study was a local one, there is no reason to expect that the greater population of hypertensive patients treated by family doctors in Poland perform their BP measurements more accurately than our subjects.

It was found that patients were less likely to make errors when measuring their BP while using automatic and wrist sphygmomanometers, which may be related to the differing levels of difficulty associated with using each device [24].

Residents of large cities made fewer mistakes than those living in small towns. It cannot be ruled out that they had greater access to specialist care, where they may have been trained to measure their BP.

Finally, it was determined that patients with a more extended history of HTN were more likely to make errors. These patients may have forgotten the correct measurement techniques they were taught previously and subsequently developed poor habits.

Should patients not measure their BP accurately, it may result in either a needed follow-up consultation not occurring or in increased strain on the healthcare system due to patients visiting their physician unnecessarily. In the latter case, there is an additional risk of patients being over-prescribed with excess or inappropriate medications, prompting further health risks.

Self-BP control has been proven to increase patient engagement and may improve adherence to HTN treatment [25–27]. However, our study has shown that patients make multiple errors as they self-measure their BP, possibly negating the potential benefits of HBPM. The results suggest a strong need for family physicians to act and educate their patients on how to measure their BP at home correctly.

HBPM training by a physician or nurse should include verifying the patient’s ability to measure their BP independently [21]. Through standardised training offered by doctors or allied healthcare personnel, the accuracy of reported BP measurements by patients improve. Therefore, it is essential to underline that repeated careful instruction of correct BP measurement technique should be provided by medical staff to patients.

We believe that a larger scale study is needed to examine the influence of concomitant diseases and patient ages [28]. Future studies should also aim to determine which patient education methods are most effective and in which patient groups.

### Conclusions

This study revealed that the majority of Polish hypertensive patients might make several errors when self-measuring their BP, significantly affecting their readings. Improperly measured BP in hypertensive patients can lead to poor disease control, increasing cardiovascular morbidity and mortality. The findings of this investigation reveal that self-administered BP measurements by patients are highly error prone. Therefore, healthcare professionals must do more to educate patients on proper BP measurement techniques specific to their device.

## Supplementary Information

**Additional file 1:** Questionnaire used in the study.

**Additional file 2:** Detailed information on sample size calculation.

## Data Availability

Data from the full survey is available upon request.

## Abbreviations

BMI: Body mass index
BP: Blood pressure
ESH: European Society of Hypertension
EQF: European Qualifications Framework
GCP: Good clinical practice
HBPM: Home blood pressure monitoring
HTN: Hypertension
OBPM: Office blood pressure measurement
