# Supplementary material for: The quality of patients’ self-blood pressure measurements: a cross-sectional study
Source: BMC Cardiovasc Disord. 2021 Nov 12;21:539. doi: 10.1186/s12872-021-02351-5 (PMC8588592; doi:10.1186/s12872-021-02351-5)
Supplement: Supplementary file 1 — Additional file 1: Questionnaire used in the study. [file 12872_2021_2351_MOESM1_ESM.pdf]

Uniwersytet Jagielloński
Collegium Medicum

Department of Family Medicine UJCM
Bocheńska 4, 31-061 Kraków

The Quality of Patients' Self-Blood Pressure Measurements.

1. **Gender** Female/ Male/ Prefer not to disclose
2. **Age**…... years
3. **Education**:
   □ elementary school □ secondary school □ high education
4. **Place of living:**
   □ Village or town of less than 50,000 inhabitant

   □ City of more than 50,000 inhabitants
5. **Time of hypertension diagnosis (years)**
6. **Family history of hypertension YES/NO**

   □ parent □ both parents □ siblings □ children
7. **Recommended hypertension medications are taken:**
   □ at all □ regularly □ irregularly
8. **Chronic comorbidities:**
   □ diabetes □ orthostatic hypotension
   □ arythmia (eg. Atrial fibrilation) □ renal insufficiency
   □ other comorbidities– what kind? ....
9. **Sources of information** (more than one answer possible)
   □ primary care doctor
   □ cardiologist
   □ other specialist
   □ nurse
   □ pharmacist
   □ patient is a medical staff
   □ internet
   □ sphygmomanometer user manual
   □ family/friends with hypertension
   □ family/friends without hypertension □ no source
10. **How were the instruction given on how to measure the blood pressure?** (more than one answer possible)
    □ oral □ written □ live demonstration □ no instruction
