# Supplementary material for: The quality of patients’ self-blood pressure measurements: a cross-sectional study
Source: BMC Cardiovasc Disord. 2021 Nov 12;21:539. doi: 10.1186/s12872-021-02351-5 (PMC8588592; doi:10.1186/s12872-021-02351-5)
Supplement: Supplementary file 2 — Additional file 2: Detailed information on sample size calculation. [file 12872_2021_2351_MOESM2_ESM.docx]

Supplementary Material 2

Detailed information on sample size calculation.

Patient sample size (n) was determined using OpenEpi software using the formula:

n = [DEFF * N * p * (1-p)] / [(d2 / Z21 - α / 2) * (N-1) + p * (1-p)],

where DEFF is the design effect, equal to 1.0; N is the population size, equal to 10,000,000 Polish citizens with HTN; p is the anticipated percentage frequency of errors made by patients during BP measurements, equal to 50%; d is the absolute error or precision, equal to 10%; α is the significance level, equal to 5%; and Z21 - α / 2 is the confidence interval, equal to 1.96.

The minimum patient sample size (n) for this study was estimated to be 97.
